# Layered Development of Visual Search Expertise in Architects

**DOI:** 10.3390/jemr19040090

**Published:** 2026-08-17

**Authors:** Spencer Ivy, K. Hudson Guttman, Trafton Drew, Jeanine K. Stefanucci

**Affiliations:** 1 Department of Philosophy, University of Missouri—St. Louis, St. Louis, MO 63121, USA; 2 Department of Psychology, University of Utah, Salt Lake City, UT 84112, USA; hudson.guttman@siu.edu (K.H.G.); jeanine.stefanucci@psych.utah.edu (J.K.S.)

**Keywords:** visual search, perceptual training, expertise, holistic visual processing, architecture

## Abstract

It is widely proposed that perceptual expertise is characterized by an increased reliance on peripheral visual information during search, as described by Holistic Visual Processing (HVP) theory. HVP predicts that experts make longer saccades to construct a holistic representation of the visual scene and facilitate target detection. Our previous work challenged this prediction by showing that expert architects made shorter, rather than longer, saccades during visual search. The present study investigated how this atypical search strategy develops by comparing expert architects, intermediate architects, and naïve observers performing the same visual search task. Intermediate architects exhibited shorter saccades than naïve observers, but not to the extent observed in experts, while demonstrating search efficiency intermediate to the two groups. Analyses of the temporal organization of search further revealed that intermediate architects had already adopted the overall structure of the expert search strategy despite not yet exhibiting expert-level reductions in saccadic amplitude. These findings suggest that perceptual expertise in architecture develops through a layered progression in which the organization of visual search precedes the full refinement of eye movement behavior. More broadly, these findings suggest that eye movement research should interpret gaze metrics such as saccadic amplitude within the context of domain-specific task demands and developmental stage, while considering the temporal organization of search as an additional window into the emergence of perceptual expertise.

## 1. Introduction

Perceptual expertise enables observers to extract task-relevant information from complex visual environments more efficiently than novices [1]. Across many domains, including medicine, sports, and security screening, experts exhibit eye movement patterns that differ systematically from those of less experienced observers, often reflecting a greater reliance on peripheral visual information during search (see [2] for a review). The reliance on peripheral vision is especially true when examining expert visual behavior in target search [3], prediction [4], and real-world scene perception [5]. These changes are reflected in several characteristic gaze behaviors, including anchoring, pivot strategies, and holistic visual processing (HVP) [6,7]. As expertise develops, observers often reduce the number of costly eye movements by extracting more information from parafoveal and peripheral vision [8,9]. Specifically, they reduce the information processing cost of generating saccades—quick eye movements from one point of fixation to another [8,9]. The present study focuses specifically on HVP because it makes explicit predictions about reliance on peripheral vision during visual search.

HVP is a search strategy common to many forms of perceptual experts, including but not limited to radiologists, ornithologists, basketball players, and TSA agents [10,11,12,13]. HVP has been proposed as a two-stage search strategy [14,15]. Observers first perform a global analysis using peripheral and parafoveal information to generate an overall representation of the scene. Attention is then directed toward candidate targets through a more focused feature search that confirms or rejects potential abnormalities [16]. HVP may lead to superior retention of information in long-term working memory [17] and the ability to select target hotspots [18]. Thus, expert perceivers are able to cognitively process visual data more easily than novices, and the visual information they process is behaviorally constrained to be more ecologically valid for their domain of expertise. These benefits lead to higher efficiency and success in visual search tasks.

The primary behavioral signifier of HVP is saccadic amplitude [3]—a measure of the distance between visual fixations. Greater saccadic amplitudes are generally interpreted as reflecting increased reliance on parafoveal and peripheral information during search [16]. Experts are able to use the extra parafoveal visual data to select targets, thereby increasing search accuracy and reducing search time [19]. The method generally used to test for these HVP search behaviors involves using a gaze-contingent viewing (GCV) window that occludes all peripheral and parafoveal vision. Because HVP is thought to rely on parafoveal and peripheral visual information, the GCV manipulation limits access to extra-foveal input, thereby disrupting the formation of a holistic representation of the visual scene [19,20,21,22,23]. By comparing search behavior in normal viewing to GCV viewing, any difference in saccadic amplitude serves as evidence for reliance on non-foveal visual data. Surprisingly, our previous work [24,25] did not support this prediction. Rather than exhibiting larger saccades, expert architects consistently produced shorter saccades than naïve observers in both unrestricted and gaze-contingent viewing.

Though surprising, these results are consistent with a new field of research suggesting that HVP strategies and reliance on peripheral visual data may not be ubiquitous across domains of expertise and may not, in fact, be a universal hallmark of perceptual learning [9,23,26,27,28]. These findings raise an important question. If architecture experts do not rely heavily on peripheral vision, how does this alternative search strategy develop? Existing studies compare experts with novices but provide little insight into the developmental trajectory of search behavior. To further uncover how perceptual search strategies develop in order to better understand why they improve the functionality and accuracy of search, we targeted intermediately experienced architects. By examining search behavior across three levels of expertise (novices, intermediates, and experts), we expected to characterize the developmental trajectory of this non-periphery-reliant search strategy.

To address this question, we compared previously published data from expert architects with a novel sample of intermediate architects and a new naïve control group. Participants completed both a domain-specific search task and a perceptually similar out-of-domain search task under normal viewing and gaze-contingent viewing (GCV) conditions, allowing us to examine whether changes in reliance on extra-foveal visual information develop with expertise and whether they generalize beyond the trained domain. By including observers at three stages of expertise, we sought to determine whether the reduced saccadic amplitudes observed in expert architects emerge gradually over the course of training or only after extensive experience. Participants completed both an out-of-domain expertise visual search task involving chest radiographs and a domain-specific perspective search task designed to share key perceptual and search demands with the radiograph task. This design allowed us to examine whether expertise-related changes in visual search were specific to the participants’ trained domain or generalized to a structurally similar task that relied on comparable underlying perceptual processes. If the expert search strategy develops progressively with experience, intermediate architects should exhibit eye movement behavior and search performance that fall between those of naïve observers and experts. Specifically, we predicted that intermediates would demonstrate greater search efficiency than naïve observers, but lower efficiency than experts, while exhibiting a partial reduction in saccadic amplitude that reflected an emerging version of the expert search strategy.

More broadly, these predictions reflect the hypothesis that acquiring expertise in architecture is accompanied by systematic changes in how visual information is sampled during search, resulting in progressively shorter saccades rather than the larger saccades traditionally associated with holistic visual processing. If supported, these findings would provide further evidence that increased reliance on peripheral vision is not a universal hallmark of perceptual expertise, but instead that visual search strategies develop in ways that are specific to the demands of a given domain [9,23,26,27,29].

## 2. Method

### 2.1. Participants

Data from 70 participants were collected from three different groups, making for 24 participants in the intermediate and naïve group, and 23 expert architects. This sample size was determined based on an a priori G*Power (ver. 3.1) analysis for a repeated-measures F-test (within factors; Cohen’s f = 0.25, α = 0.05, four measurements, assumed correlation among repeated measures = 0.5), which indicated a total sample of 24 for power of 0.8. Because the present design adds a between-subjects factor, we conducted a sensitivity analysis confirming that the final sample (N = 70) provides 80% power to detect a group × viewing condition interaction of f = 0.19 (η^2^p = 0.035) and a group main effect of f = 0.33 (η^2^p = 0.1). The data from the expert architects were previously published in Ivy et al. [24,25]. Experts in architecture (*n* = 27, 7 female and 22 male) were professionals who held a bachelor’s degree in architecture, a master’s degree in architecture (or both), or a license to practice architecture. Architecture experts were, on average, 42 years old (*SD* = 11.63), with an average of 19 years of experience (*SD* = 12.12). Architecture experts had no experience or formal training with medical imaging. Four architects were excluded from analysis due to poor eye tracking (leading to 23 included in the final sample). Participants with bifocals were excluded from analysis due to poor calibration from the eye tracker; poor eye-tracking calibration was also the reason some intermediate and naïve participants were excluded. Expert architects were recruited from three different firms and were each given $50 for their participation. Intermediates in architecture (*n* = 24, 17 female, 7 male) were either an undergraduate or master of architecture student at the University of Utah. This sample of intermediates in architecture was, on average, 24 years old (*SD* = 6.19) and had an average of 3 years of experience (*SD* = 1.45). None of the intermediate architects had any experience or formal training with medical imaging. Intermediate architects were given $50 for their participation. Naïve participants consisted entirely of undergraduate psychology students (*n* = 24, 19 female, 5 male) from the University of Utah. Naïve participants were, on average, 20 years old (*SD* = 2.27) and had no experience, degrees, or formal training in art, architecture, or medical imaging. Naïve participants received psychology course credit for their participation. Through the onboarding process, participants were asked whether they needed glasses or contacts for vision and were requested to use these upon participating in the tasks. Thus, all participants had normal or corrected-to-normal vision. All procedures were reviewed and approved by the University of Utah Institutional Review Board and followed the tenets of the Declaration of Helsinki.

### 2.2. Materials

Gaze was recorded with an EyeLink Plus 1000 eye tracker, which sampled at 1000 Hz and resolved gaze position to within 0.5° of visual angle. A laptop running EyeLink OS (Version 5.15) controlled the tracker, while a separate desktop PC delivered the experiment through the Psychophysics Toolbox in MATLAB (ver. R2017a) [30,31,32]. A headrest was used to stabilize participants’ heads in order to reduce participant fatigue and have consistent calibration throughout the trials. The headset was adjusted for height and posture by the participants themselves before the trials began. In the trials, consistently sized images of 28.3 cm × 28.3 cm were used, subtending a visual angle of 24.6° in both directions. Images were presented without pixelation on a 21-inch monitor with a 1920 × 1080 resolution. In order to simulate reading rooms for radiologists and provide the best lighting for eye tracker calibration, room lights were turned off and a single lamp in the corner was dimmed. Certain trials involved a gaze-contingent window, which was used to limit the vision of the image and save a single point of fixation. A bivariate Gaussian circle (half-width at half-maximum measured 1.8°) was followed by the unobstructed area of the gaze-contingent window slowly fading to gray. At 1.8° from the point of fixation, the gaze-contingent area was partly gray and became darker while moving to full opaque at 4.9°. The GCV window size and its relative fade-to-gray period were chosen to be consistent with our prior research [24,25]. The parameters of the GCV window were set to meaningfully restrict extra-foveal input and ensure the restriction of the HVP search strategy.

Twenty-two single-view chest radiographs were used as the radiology images in the PA projection from the Japanese Society of Radiological Technology database. Half of these radiographs pictured a lung with a cancerous tumor. The radiograph images varied in degree of search difficulty according to the subtlety of targets relative to each image. The rated degree of difficulty was based upon the work of Shiraishi et al. [33] which categorized images on a scale from 1 (most subtle/most difficult) to 5 (least subtle/least difficult). Equal groupings of both easier and more difficult images were distributed within each block of the experiment, with an average difficulty rating of 3. However, each block included images from both upper and lower limits of Shiraishi’s scale.

**Figure 1:**
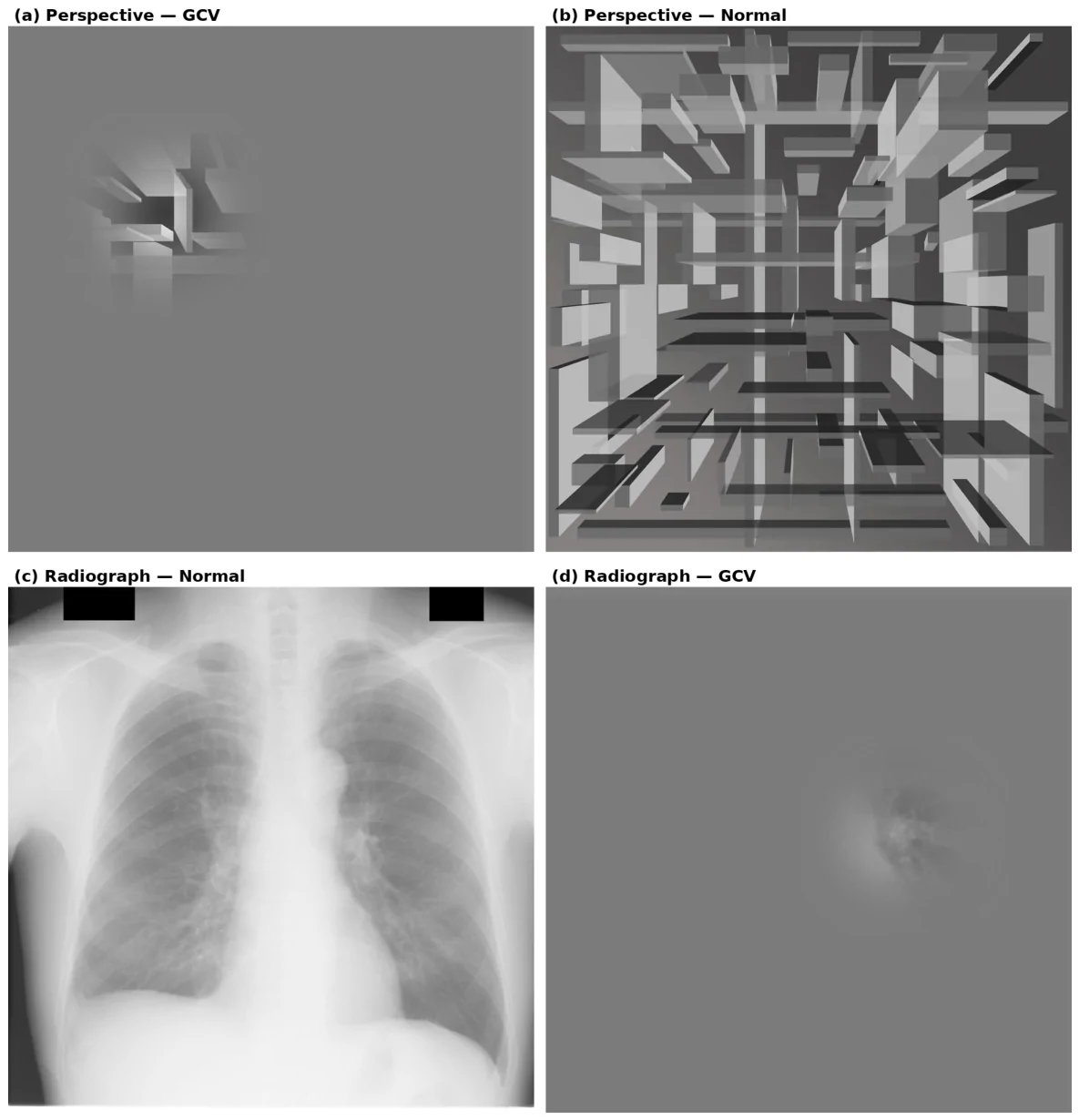
Example stimuli used in the experiment. (**a**) Zero-point perspective image under gaze-contingent viewing (GCV), showing the restricted viewing window. (**b**) The same perspective image under normal viewing. (**c**) Chest radiograph under normal viewing. (**d**) Chest radiograph under GCV.

Using the 3DS Max software (ver. 2017) suite, we created twenty-two grayscale perspective images, each containing between 40 and 150 geometric shapes positioned in zero- or one-point perspective relative to one another. The edges of these shapes ran in parallel and, had the shapes been in motion, would have converged on a horizon point centered on the display (see Figure 1). In eleven of the images, one shape was set out of perspective with the rest and served as the target. These zero-point perspective images were used to test for architects’ visual expertise patterns and search behaviors. The zero-point perspective model was developed with architects as a set of stimuli that uphold the forms of images architects examine and search as part of their practice. The task therefore drew on the visual skills used to judge holistic congruence and spatial parallelism in 3D digital design. These stimuli were validated in prior studies, where we demonstrated that they are able to discern search behaviors associated with expertise across domains [24,25]. As in the radiology images, zero-point perspective images were ranked by target subtlety and distributed equally across the counterbalanced participant groups, in order to include challenging and easy search images.

### 2.3. Procedure

Participants gave informed consent and then adjusted the eye-tracking headrest for comfort. Calibration and validation followed, using a 9-point procedure accepted only at error below 0.5° of visual angle. Participants were then informed that they would search for targets in two image types (radiographs and zero-point perspective images) under two viewing conditions (normal and gaze-contingent). The normal viewing condition was explained to the participant as typical observation of a computer monitor, where the entire screen and image on the screen are visible. The gaze-contingent condition was explained to participants as a small window that would follow the point of their visual fixation. This was described as like “pointing a flashlight at a wall in a dark room,” with the gaze-contingent window being the small circle of illumination a flashlight would offer. Window position was driven by the eye tracker, which tracked gaze and refreshed the display at 120 Hz. Instructions differed by image type: in radiographs, participants searched for tumors or other signs of cancer; in the zero-point perspective images, for the box that was out of perspective with the others. Participants were told that a target was present on half of the trials. When no target could be found, they indicated this by clicking a box at the left edge of the screen.

In order to familiarize participants with the targets involved with each image type, participants were presented with trial images before each block of the experiment began. Eight practice trials were administered in total, four per image type. Every practice image contained a target, and its location was revealed to participants during practice only. Viewing condition was then blocked within each image type and its order counterbalanced across participants. Each subsequent experimental block presented 22 trials drawn from one image type (zero-point perspective or radiograph), divided evenly into the two viewing conditions (normal viewing and GCV), resulting in 11 images for each condition. After finishing the first 22-image block, participants completed the same task with the remaining image type. Image type and viewing condition were counterbalanced across participants, and image order was randomized within each counterbalancing group. No image was repeated for a participant, either within or across blocks. Once all 44 images had been searched, the session closed with a post-performance interview collecting qualitative reports of search confidence and of the strategies employed in each task and condition. Qualitative results of the interviews with the expert architects are reported in a previous study [25]. All groups were studied on the same equipment with the same software version, the same parameters, and under comparable laboratory conditions. To confirm that neither practice nor fatigue was driving our reported group differences, we added viewing-condition order as a between-subjects factor to the saccadic amplitude analysis. For the perspective images, order neither affected saccadic amplitude overall (F(1,64) = 0.04, *p* = 0.85 ), nor interacted with expertise group (F(2,64) = 0.04, *p* = 0.96), indicating that the differences we report between naïve, intermediate, and expert groups are not attributable to the order in which the viewing conditions were encountered.

## 3. Results

The results are organized by image type and include a comparison of effects within and across the three groups. The data were analyzed using mixed-effect analyses of variance (ANOVAs). For data comparing the intermediate, naïve, and expert populations, we report omnibus ANOVAs consisting of the three groups and the relevant viewing conditions. These include comparisons of each group with one another (expert, intermediate, naïve) as well as viewing condition (GCV or normal viewing) and the interaction of those two factors with one another. When making individual comparisons across groups within a condition or within a group across conditions, *t*-tests were performed. When results to these *t*-tests were either significant or insignificant but meaningful to interpreting the data, *p*-values are reported in order to demonstrate the direction (or lack) of relevant effects. Statistics for these data are presented below in Table 1 and Table 2. As with our previous studies [24,25], we found that the data for accuracy of target search closely tracked those for *d*-prime (*d*′) and so will only report *d*′. Finally, to simplify the presentation of these data and to track the hypotheses we set out to investigate, target-absent trials were dropped due to the fact that they were not central to the research question. They were also dropped because they involved a large final saccade to the “no target present” box that systematically inflated the late-trial saccadic amplitude. As we were interested in the natural process of search, excluding these absent trials kept saccadic amplitude as ecologically valid as possible.

### 3.1. Behavioral Results for Perspective Images

**Figure 2:**
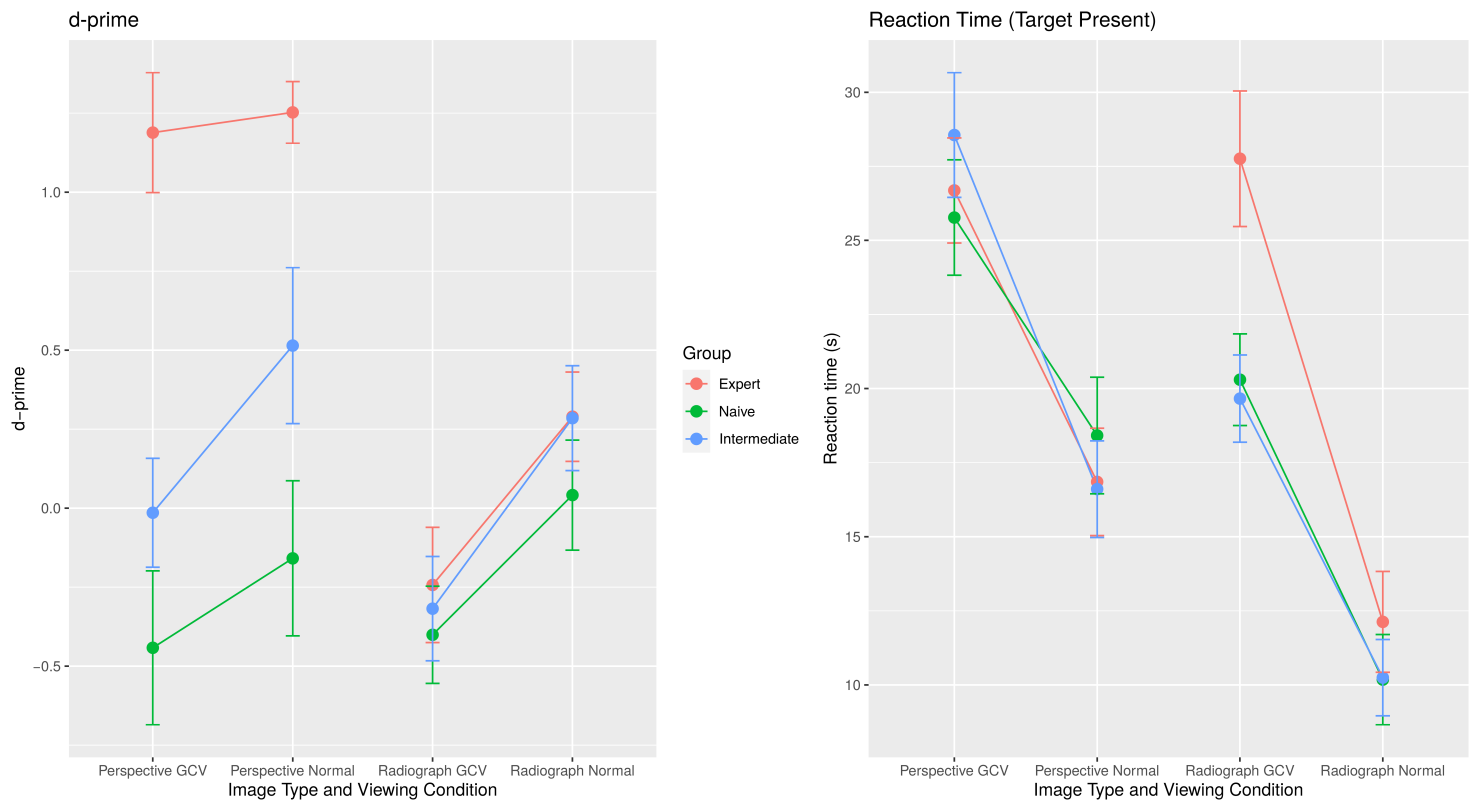
*d*′ (**left**) and reaction time (**right**) for target-present trials across image type, viewing condition, and expertise group. Error bars represent ±1 SE. Only target-present trials are reported.

*d*′ (see Figure 2) is a measure of probability concerning participants’ likelihood to correctly or incorrectly identify the presence or absence of a target by weighing the probability of false alarms against that of correct designations of a target’s presence [34]. There was a main effect of observer group [*F*(2,67) = 27.69, *p* < 0.01, $\eta_{G}^{2}$ = 0.28], with the expert group being significantly more sensitive to targets than both the intermediates (*p* < 0.01) and the naïve group (*p* < 0.01). The intermediates were also more sensitive to targets than the naïve group (*p* < 0.01). Interestingly, the difference between normal viewing and GCV *d*′ for the experts was not significant (*p* = 0.39). Likewise, the difference between the normal viewing and GCV condition for the naïve group was also insignificant (*p* = 0.21). However, the difference between viewing conditions for the intermediate group was significant (*p* = 0.04). What these data indicate, in addition to the fact that the experts were far more sensitive to targets than the naïve group, is that the expert group was equally likely to correctly select targets in either GCV or normal viewing, while, in contrast, the naïve group were equally likely to be insensitive to targets across conditions. It was only the intermediates for whom the GCV condition significantly lowered their sensitivity to targets relative to normal viewing.

With respect to reaction time, there was no significant difference between the three groups [*F*(2,67) = 0.07, *p* = 0.93, $\eta_{G}^{2}$ < 0.01]. However, there was a significant difference in the reaction time for viewing condition [*F*(1,67) = 54.15, *p* < 0.01, $\eta_{G}^{2}$ = 0.22], in that all three groups exhibited slower reaction times to identify targets under gaze-contingent conditions. Further, there was no interaction of group with viewing condition [*F*(2,67) = 1.04, *p* = 0.36].

### 3.2. Behavioral Results for Radiograph Images

For radiograph images, there were no significant differences between groups in *d*′ [*F*(2,67) = 0.73, *p* = 0.49, $\eta_{G}^{2}$ = 0.01]. There was, however, a significant effect of viewing condition across all three groups’ *d*′ [*F*(1,67) = 17.37, *p* < 0.01, $\eta_{G}^{2}$ = 0.10] due to the fact that GCV reduced the sensitivity of all participants. This effect was likely driven by the fact that all three groups are equally inexperienced when searching for abnormalities in radiographs and that the GCV window makes a challenging task even more challenging for inexperienced viewers. There was no interaction of group and viewing condition [*F*(2,67) = 0.14, *p* = 0.87, $\eta_{G}^{2}$ < 0.01].

There was a significant difference across the three groups’ reaction times for radiograph images [*F*(2,67) = 3.60, *p* = 0.033, $\eta_{G}^{2}$ = 0.075], such that the experts were slower than the other two groups. There was also a significant difference between the three groups in viewing condition, [*F*(1,67) = 143.87, *p* < 0.01, $\eta_{G}^{2}$ = 0.35] in that the experts’ reactions were much faster on normal viewing than in GCV compared to the intermediate (*p* < 0.01) and the naïve (*p* < 0.01) groups. Finally, there was also a significant interaction of group and viewing condition, [*F*(2,67) = 3.99, *p* = 0.02, $\eta_{G}^{2}$ = 0.03]. The expert group took a significantly longer amount of time to react to a target’s presence than both the intermediate group (*p* = 0.02) and the naïve group (*p* = 0.04). There was no significant difference between the reaction times of the naïve group and intermediates (*p* = 0.88).

### 3.3. Eye Tracking Results for Perspective Images

**Figure 3:**
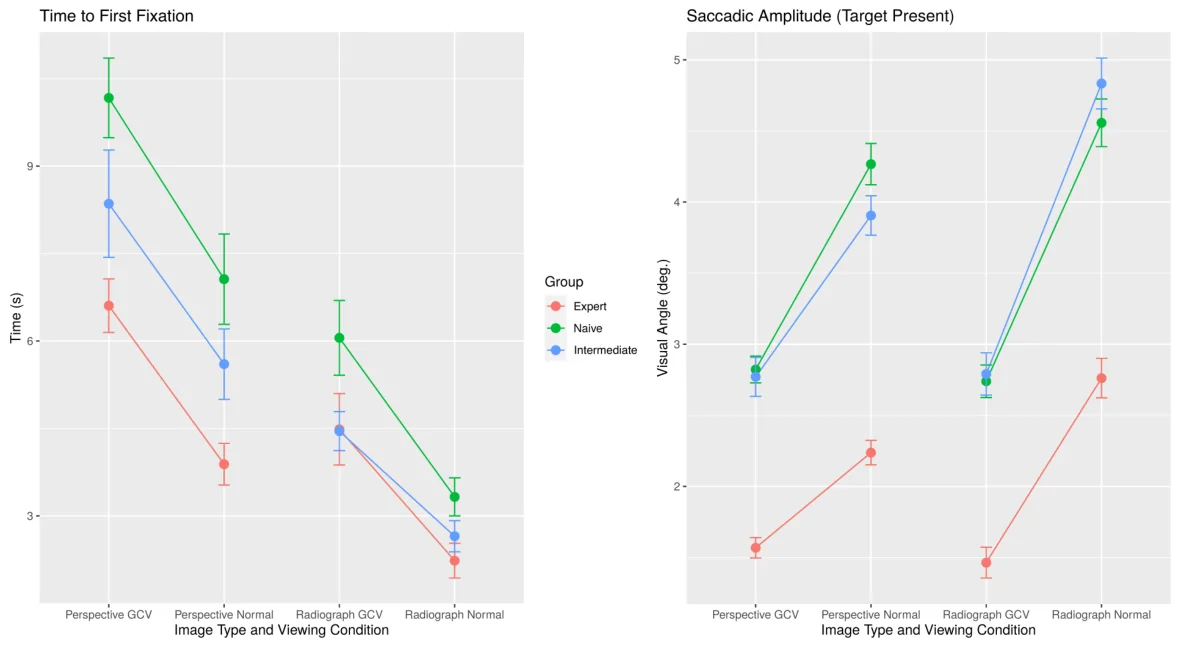
Time to first fixation (**left**) and saccadic amplitude (**right**) for target-present trials across image type, viewing condition, and expertise group. Error bars represent ±1 SE. Only target-present trials are reported.

There was a significant difference in the saccadic amplitude (see Figure 3) for group [*F*(2,67) = 83.20, *p* < 0.01, $\eta_{G}^{2}$ = 0.62], such that the experts exhibited shorter saccadic amplitudes than the intermediate (*p* < 0.01) and naïve (*p* < 0.01) groups. There was also a main effect of viewing condition on saccadic amplitude [*F*(1,67) = 191.12, *p* < 0.01, $\eta_{G}^{2}$ = 0.49] in that GCV universally reduced the amplitude of saccades across groups. Finally, the interaction of group with viewing condition was significant, [*F*(2,67) = 7.91, *p* < 0.01, $\eta_{G}^{2}$ = 0.07]. As expected, these data indicate that experts exhibited much shorter saccades than both the intermediates and the naïve group and were less affected by GCV. Additionally, the intermediate group utilized a lower saccadic amplitude in the normal viewing condition than the naïve group (*p* < 0.01). However, interestingly, there was no significant difference between the naïve group and intermediates in the GCV condition (*p* = 0.17).

Time to first fixation measures the amount of time from a trial’s beginning to the time at which the participant’s gaze first fixates upon a target. In contrast, decision time measures the time between the first fixation on the target and when a final decision is made and the target is reported. We hypothesized that experts would be the quickest with respect to both first fixation and decision times, with intermediates following and the naïve group being the slowest. There was a main effect of group on first fixation [*F*(2,67) = 13.60, *p* < 0.01, $\eta_{G}^{2}$ = 0.15]. Upon comparing the three groups, our hypothesis was confirmed; experts enjoyed the quickest first fixation time, significantly faster than the intermediates (*p* = 0.01). Similarly, intermediates were significantly faster than the naïve group (*p* = 0.02).

Contrary to our hypothesis that each group would have significantly different decision times, there was only a significant difference in decision time across viewing conditions [*F*(1,67) = 44.75, *p* < 0.01, $\eta_{G}^{2}$ = 0.19]. This is because, in the GCV condition, intermediates were slower to decide than the experts (*p* < 0.01) and the naïve group (*p* < 0.01). Although faster than the intermediates, the naïve group was slower to decide than the experts in the GCV (*p* < 0.01). There was no significant difference in the decision time of all three groups in the normal viewing condition (*p* = 0.06).

### 3.4. Eye Tracking Results for Radiograph Images

For radiograph images, there was a significant difference in saccadic amplitude by group [*F*(2,67) = 65.06, *p* < 0.01, $\eta_{G}^{2}$ = 0.54], in that the experts exhibited a significantly shorter amplitude than the intermediates (*p* < 0.01) and the naïve group (*p* < 0.01). Interestingly, there was no significant difference between the naïve group and intermediate group’s saccadic amplitudes in radiograph images (*p* = 0.35). There was also a significant effect of viewing condition across the three groups’ saccadic amplitudes [*F*(1,67) = 257.92, *p* < 0.01, and $\eta_{G}^{2}$ = 0.61]. This is because, mirroring the perspective task, the GCV condition universally lowered saccadic amplitude in all groups. Additionally, there was a significant interaction of group and viewing condition [*F*(2,67) = 4.08, *p* = 0.02, $\eta_{G}^{2}$ = 0.05]. Because this interaction was significant for the experts in comparison to intermediates (*p* = 0.02) and the naïve group (*p* < 0.01), it is likely that the expert architects maintained a consistent search strategy across tasks.

There was a significant main effect of time to first fixation across the three groups [*F*(2,67) = 3.89, *p* = 0.02, $\eta_{G}^{2}$ = 0.07], such that the naïve group took longer than the intermediates (*p* = 0.03) and the experts (*p* = 0.03). There was no significant difference in the time to first fixation in radiographs when comparing the experts to the intermediates (*p* = 0.6). There was also a significant effect of viewing condition on time to first fixation across the three groups [*F*(1,67) = 59.11, *p* < 0.01, and $\eta_{G}^{2}$ = 0.22], which was driven by the naïve group who were more affected by GCV than the intermediates (*p* < 0.01) and experts (*p* < 0.01). Here again, there was no significant difference in the performance of the intermediate compared to the expert group. There was, however, no significant interaction between viewing condition and group [*F*(2,67) = 0.84, *p* = 0.43].

The decision time for all three groups was remarkably similar, leading to no significant effect based on group [*F*(2,67) = 0.02, *p* = 0.98, and $\eta_{G}^{2}$ < 0.01]. The decision times for all three groups, however, were significantly affected by viewing condition [*F*(1,67) = 105.09, *p* < 0.01, $\eta_{G}^{2}$ = 0.32] due to the fact that each group made decisions much faster in the normal viewing condition. Finally, there was no interaction of group and viewing condition on radiograph decision time [*F*(2,67) = 0.16, *p* = 0.85, $\eta_{G}^{2}$ < 0.01].

### 3.5. Continuous Search Trends

**Figure 4:**
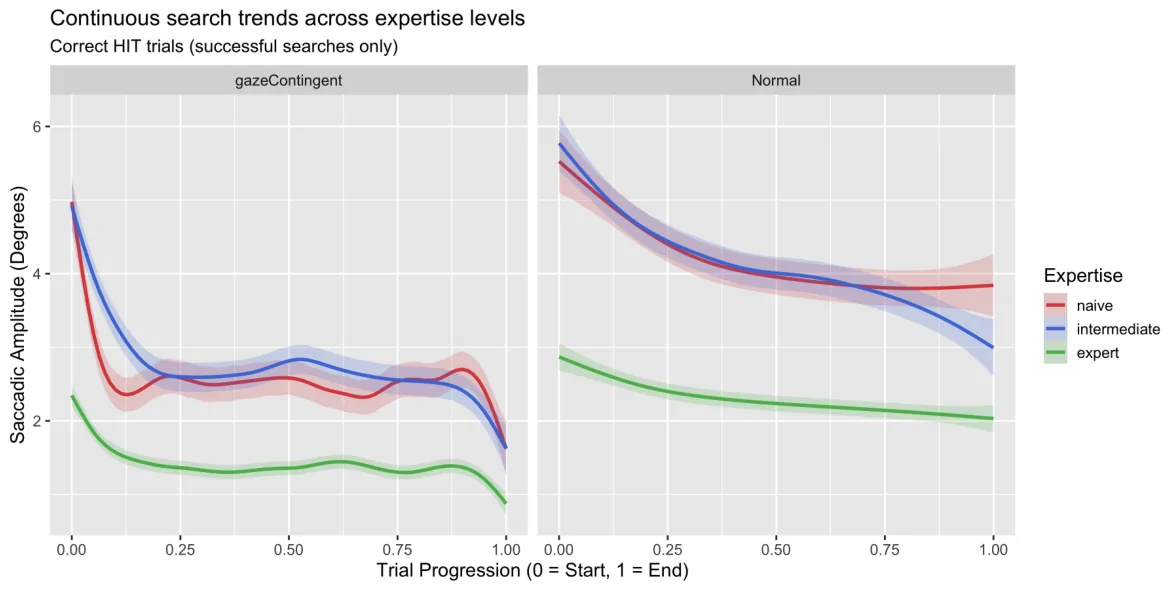
Continuous search trends for correct hit trials. On the y-axis, saccadic amplitude (degrees) is plotted against the x-axis trial progression as a normalized function (0 = start, 1 = end). The plots include each expertise group (red = naive, blue = intermediate, green = expert), separated by viewing condition (gaze-contingent, (**left**); normal, (**right**)). Shaded regions represent 95% confidence intervals from generalized additive model fits.

To examine how saccadic amplitude unfolds across the course of a search, we normalized each saccade’s position within its trial to a 0–1 scale (0 = first saccade, 1 = last saccade) and fit them to Generalized Additive Models (GAMs). These analyses allow us to test not only whether overall levels of saccadic amplitude differ between groups and conditions, but also how saccadic amplitude changes dynamically throughout the trials (see Figure 4). Examining these temporal dynamics provides a more sensitive test of our overarching hypothesis that expertise is associated with changes in visual search strategy, as differences between novices, intermediates, and experts may emerge or evolve over the course of a search rather than being captured by average saccadic amplitude alone. We report the analysis of correct hit trials only as this subset avoids a motor artifact present in miss and correct-rejection trials. On those trials, participants executed a large saccade to the “no target present” button at the edge of the screen, artificially inflating amplitude at the end of the trial for all three groups.

We fit all the GAM models in R using the mgcv package (ver. 1.9-4) [35,36]. Saccadic amplitude was modeled as a function of expertise group, viewing condition, and their interaction, along with a separate smooth curve of trial progression for each combination of group and viewing condition. These curves were smoothed by cubic regression splines with shrinkage estimated by restricted maximum likelihood with a basis dimension of k = 20. The k-value operates as a ceiling on how much a curve is permitted to bend, and our fitted curves fell well beneath that ceiling (all edf ≤ 11.11), with no evidence of unmodeled structure (k-index = 1.01, *p* > 0.72 for all six curves). Refitting every model to k = 40 changed the effective degrees of freedom by no more than 0.63 and left all conclusions about curve shape intact, indicating that k = 20 and the shrinkage were sufficient to guard against overfitting. Concurvity among the smooth terms was negligible (the highest was ≤0.004). Finally, we fit these models at the level of individual saccades without a random effect for each participant. Because our central claims rest on the absence of a difference in curve shape between the intermediate and expert groups, any inflation of significance arising from non-independence between participants would work against these conclusions rather than in their favor.

All three groups exhibited a steep initial decline in saccadic amplitude during approximately the first 10–15% of each trial, likely reflecting an orienting response before a search strategy fully engages. After this initial drop, the groups diverged markedly. Experts maintained a consistently flat and low saccadic amplitude throughout the remainder of each trial in both viewing conditions (approximately 1.1–2.2°), with low effective degrees of freedom in both normal viewing (edf = 2.01, *F* = 1.34) and gaze-contingent viewing (edf = 3.74, *F* = 1.74), indicating a stable search strategy across viewing conditions. In contrast, the naïve and intermediate groups showed more dynamic shifts in their respective search strategies across viewing conditions. Particularly for normal viewing, both groups began with high amplitudes that gradually declined but never approached expert levels. Interestingly, in GCV, intermediates exhibited slightly higher amplitudes than the naïve group (*p* < 0.001), which demonstrates a reversal from the trend observed in normal viewing. Nevertheless, the naïve and intermediate curves converge to similar amplitudes (∼2.5°) after the initial orienting saccades. Experts remained substantially lower (∼1.5°), suggesting that the expert search strategy is internalized and operates independently of viewing conditions.

Finally, pairwise comparisons of the continuous trend’s curve shape reveal a pattern consistent with the developmental trajectory of search behavior at different stages of expertise. The shape of the expert curve differed significantly from the naïve curve in both the normal viewing condition (edf = 1.19, *p* = 0.001) and GCV (edf = 0.25, *p* < 0.001), but the expert and intermediate curve shapes were not significantly different in either condition (normal: *p* = 0.085; GCV: *p* = 0.175). Meanwhile, the intermediate curve shapes differed significantly from the naïve group in both conditions (normal: edf = 0.84, *p* = 0.005; GCV: edf = 4.64, *p* < 0.001). Taken together, these comparisons suggest that while the intermediates have not yet achieved the low overall amplitude characteristic of expertise, they have already begun to adopt an expert-like pattern of search. In other words, the form of the search strategy appears to develop before the magnitude of saccadic suppression is fully realized. This dissociation reinforces the interpretation that learning to search like an architect involves a progressive reorganization of visual exploration in which the initial strategies are acquired relatively early in training, while the full suppression of saccadic amplitude continues to deepen with years of experience.

## 4. Discussion

The purpose of this study was to better understand the visual search behavior of architects by examining how reliance on peripheral visual data may change with increasing degrees of expertise. To this end, our primary hypothesis was that intermediately experienced architects, like experts, would suppress the amplitude of their saccades—though likely not to the same degree as the experts given the difference in training time. The results of this study supported our hypothesis. We did, however, expect the intermediates to suppress their saccadic amplitude to a greater degree than we found. Nevertheless, the significant degree of reduction measured in the intermediates’ saccadic amplitude not only supports the hypothesis, but these data are also consistent with our findings from Ivy et al. [24]. Namely, contrary to conventional wisdom, visual expertise in architecture is correlated with very little reliance upon peripheral visual information—and our findings suggest that this reduced reliance upon peripheral visual information develops over time with training in the field. At least with respect to architecture as a domain of visual expertise, the eye-movement characteristics traditionally associated with HVP appear not to play a prominent role in search accuracy and efficiency and are inversely proportional to an architect’s amount of experience. In other words, expert architects appear to be more efficient at in-domain visual search tasks precisely because they reduce the amplitude of their saccades and thus rely less upon peripheral visual data.

Despite exhibiting shorter saccades, both the expert and intermediate architects were more accurate and efficient than the naïve group, with experts reaching targets most quickly and intermediates performing between the two groups. These findings are consistent with our hypothesis that perceptual expertise in architecture develops alongside progressively reduced saccadic amplitudes rather than increased reliance on peripheral visual information. One prediction, however, was not supported. We expected intermediates to make decisions as quickly as experts, yet under GCV the experts made decisions most rapidly, whereas intermediates were the slowest. Notably, overall reaction times did not differ between groups, suggesting that the observed differences reflect specific aspects of search and decision processes rather than differences in the total time spent completing each trial.

Like the other measures, we expected intermediate architects to be more accurate (as measured by *d*′) than the naïve group, but less accurate than the experts, and this prediction was supported. The experts exhibited the highest target sensitivity, followed by the intermediates and then the naïve observers. However, the overall pattern of results did not support our expectation that all measures of perceptual expertise would develop proportionally with experience [37,38,39]. Although the intermediates consistently performed between the naïve and expert groups on measures of search accuracy and efficiency, the reduction in saccadic amplitude was much more pronounced in the experts. In other words, improvements in search efficiency and target sensitivity appeared to develop relatively gradually, whereas the marked reduction in saccadic amplitude emerged later in expertise. At face value, this pattern suggests that the development of perceptual expertise in architecture is not strictly proportional across all behavioral measures. Rather, it raises the possibility that changes in eye movement behavior and improvements in search performance follow partially distinct developmental trajectories.

The continuous search trend analysis provides a more nuanced view of how expertise develops. Rather than suggesting that perceptual learning progresses unevenly, the GAM analyses indicate that search expertise develops in a layered manner. The intermediately experienced architects already share the shape of the expert curve, yet the intermediates lack the overall suppression of saccadic amplitude exclusive to the experts. Therefore, although intermediate architects had not yet achieved the markedly reduced saccadic amplitudes of the experts, the temporal organization of their search behavior closely resembled that of the expert group and differed from that of the naïve observers. This dissociation suggests that the overall structure of an expert search strategy is acquired before the full refinement of eye movement behavior. In other words, architects appear to first learn the organization of an effective search strategy and only later, through continued experience, progressively reduce their saccadic amplitudes. This layered pattern of development extends existing theories of perceptual learning by suggesting that changes in the temporal organization of search may precede changes in aggregate eye movement measures.

The dissociation between the pattern and magnitude of saccadic search behavior carries implications for how we understand the development of perceptual expertise more broadly. Theories of perceptual learning that emphasize progressive, proportional gains [37,38] would predict that intermediate searchers should exhibit search behaviors that are proportionally intermediate in all respects, including both the organizational dynamics of a search and the overall level of saccadic amplitude. Yet what we observed is that the intermediates have reorganized how they search well before they have fully constrained how far their eyes move between fixations. One possible interpretation of these findings, though speculative, is that the organizational structure of the search strategy is learned through formal training and exposure to architectural stimuli (e.g., assignments and lessons in a master’s program), whereas the deeper suppression of saccadic amplitude may depend upon prolonged, chronic engagement with domain-specific material experienced over many years of professional practice. If this interpretation is correct, it would suggest that the perceptual system first learns what to do before spending considerably more time refining how to execute like an expert. Testing for such a finding could include a longitudinal study of the same set of architects from their education through their early career to see if the trend holds, or otherwise to recruit several groups of subjects with far fewer years of experience difference between them.

An important implication of these findings is that expertise may be reflected not only in search efficiency, but also in the stability of search behavior across changing viewing conditions [40,41]. Although the experts were only modestly more accurate and efficient than the intermediate architects, their search behavior was considerably less affected by the GCV manipulation. This interpretation is broadly consistent with recent work by Delucchi et al. [42], who reported that architects exhibited more consistent and focused visual search patterns than naïve observers across a range of scene types. Together, these findings suggest that prolonged architectural training may refine visual search in ways that extend beyond improvements in efficiency alone, producing search strategies that remain stable despite changes in the availability of visual information. Such robustness across viewing conditions may represent an additional dimension of perceptual expertise that complements traditional measures of accuracy and search efficiency.

The continuous trend data also lend additional support to the reliability interpretation. Across both viewing conditions, the expert saccadic amplitude remained substantially flat, indicating a stable pattern of search behavior following their initial orienting response. This stability across conditions was not exhibited by the naïve and intermediate groups, who showed more dynamic fluctuations throughout trials, especially in normal viewing. The similarity of the experts’ saccadic amplitude profiles across normal and GCV viewing suggests that their visual search strategy was relatively stable despite changes in the availability of peripheral visual information. This condition-invariant pattern further distinguishes the experts from the intermediate and naïve groups and is consistent with the interpretation that reduced saccadic amplitudes support a visual search strategy that is both efficient and robust across viewing conditions.

These findings raise an important question. If progressively shorter saccades are not associated with large gains in search efficiency, what function do they serve? To recap, experts not only had a much lower and condition-invariant saccadic amplitude profile relative to the other groups, but their performance drop was smaller between GCV and normal viewing conditions. Accordingly, we believe that the significantly lower saccadic amplitudes of the experts combined with the condition-invariance of their search trends indicate consistency and reliability through variable or unfavorable search conditions. Although the experts were only relatively more accurate and efficient than the intermediates, they were less affected by the GCV condition. Consequently, it appears that the architects suppress saccadic amplitude not only for the sake of better search, but also for the sake of more consistent and reliable search.

Taken together, these findings suggest that progressively reduced saccadic amplitudes are a characteristic of developing expertise in architectural visual search. More broadly, they challenge the common assumption that increased reliance on peripheral vision is a universal feature of perceptual expertise. Instead, our findings support a more domain-specific account in which eye movement strategies reflect the perceptual demands of the task rather than a single expert search pattern. Accordingly, evaluations of visual expertise should consider not only accuracy, efficiency, and eye movement behavior, but also the reliability and consistency of search performance across varying viewing conditions.

Several limitations should be considered when interpreting these findings. First, the intermediate group included participants with varying levels of architectural training, ranging from undergraduate to master’s students. However, all had successfully completed architecture-specific entrance requirements and were enrolled in the same competitive program. Second, the three groups had very slightly different numbers of participants due to eye-tracking calibration failure. Third, we recruited a fairly balanced male-female ratio of experts, but a much larger female-dominant population of intermediates because of differences in enrollment in our undergraduate and master’s programs. Nevertheless, we do not think that gender plays a significant role in perceptual expertise given the stability of the reported search trends between the groups. Finally, although our perspective task does not directly replicate the day-to-day work of architects, it was designed to isolate the visual search processes common to architectural practice. The consistent pattern of results observed across both expert and intermediate architects suggests that the task captured meaningful aspects of domain-specific visual expertise. Future work should examine whether similar patterns emerge in more ecologically valid architectural tasks, such as blueprint review, CAD modeling, or collaborative design evaluation.

An important unanswered question is why some domains of visual expertise appear to benefit from larger saccades whereas others exhibit the opposite pattern. We suggest that the most fruitful way forward in answering this question would be to measure the difference between search behavior and search goals. Perhaps the goals of visual search for radiologists and medical image reviewers are different than that of architects. Whereas viewing a radiograph requires one look for either normalcy or aberrancy, architectural perception involves creative insight into perspective and the presence of structure within façade. Perhaps these different goals drive divergences in physical search behaviors—but through the mastery of either form of perceptual expertise, there should always be increased search efficiency, accuracy, and reliability. To find out how and why divergent goal structures of individual domains of expertise can yield unique search behaviors while always increasing efficiency and accuracy is a fascinating and important direction for future research in perceptual expertise.

## 5. Conclusions

Our findings suggest that perceptual expertise in architecture is characterized by progressively reduced saccadic amplitudes rather than increased reliance on peripheral vision. Importantly, this development appears to be layered: intermediate architects have already acquired the organizational structure of the expert search strategy, yet continue to refine their eye movements with experience. These results challenge the assumption that the eye-movement characteristics traditionally associated with holistic visual processing are universal markers of perceptual expertise. Instead, they suggest that visual search strategies develop in ways that are specific to the perceptual demands of a given domain, highlighting the need for broader theories of visual expertise that accommodate multiple developmental trajectories. More broadly, these findings underscore the importance of considering domain-specific task demands when interpreting eye movement behavior, suggesting that common gaze metrics such as saccadic amplitude should not be treated as universal markers of expertise but instead understood within the context of the perceptual and cognitive demands of a given task.

## Figures and Tables

**Table 1:** Perspective Images. Means and standard deviations by observer group, viewing condition, and target presence.

		Experts		Intermediates		Naïve	
Measure		GCV	Normal	GCV	Normal	GCV	Normal
Accuracy	*M*	0.61	0.68	0.45	0.62	0.43	0.44
	*SD*	0.18	0.14	0.18	0.17	0.25	0.23
*d*′	*M*	1.19 *	1.25 *	−0.01 * ^†^	0.51 * ^†^	−0.44 *	−0.16 *
	*SD*	0.93	0.48	0.84	1.21	1.19	1.20
SA	*M*	1.57 *	2.24 *	2.77	3.90 *	2.82	4.27
	*SD*	0.35	0.42	0.67	0.68	0.46	0.71
RT (s)	*M*	26.69	16.85	28.56	16.61	25.77	18.42
	*SD*	8.69	8.88	10.31	7.99	9.54	9.64
TTFF	*M*	6.61 *	3.89 *	8.36 *	5.60 *	10.17 *	7.06 *
	*SD*	2.25	1.75	4.51	2.96	3.34	3.79
Decision time	*M*	14.69 *	11.05	20.44 *	11.26	17.82 *	11.74
	*SD*	4.44	5.98	8.28	7.85	7.67	6.88
Scan path ratio	*M*	14.98	13.00	17.76	15.17	13.96	13.20
	*SD*	7.43	5.31	15.58	12.58	9.68	9.08

Note. All measures are for target-present trials. Averages were computed within participants, then across participants. Statistically significant figures reported here and below are marked with an asterisk (group differed from the other groups, *p* < 0.05); a dagger (^†^) marks a significant GCV vs. normal viewing difference within that group. Accuracy and scan path ratio are descriptive only. SA = saccadic amplitude; RT = reaction time; TTFF = time to first fixation.

**Table 2:** Radiograph images. Means and standard deviations by observer group, viewing condition, and target presence.

		Experts		Intermediates		Naïve	
Measure		GCV	Normal	GCV	Normal	GCV	Normal
Accuracy	*M*	0.39	0.50	0.42	0.53	0.40	0.52
	*SD*	0.22	0.13	0.24	0.18	0.20	0.20
*d*′	*M*	−0.24	0.29	−0.32	0.28	−0.40	0.04
	*SD*	0.89	0.69	0.81	0.81	0.75	0.85
SA	*M*	1.46 *	2.76 *	2.79	4.83	2.74	4.56
	*SD*	0.53	0.68	0.73	0.88	0.56	0.82
RT (s)	*M*	27.76 *	12.13 *	19.66	10.25	20.30	10.18
	*SD*	11.21	8.33	7.22	6.31	7.57	7.45
TTFF	*M*	4.48	2.23	4.45	2.65	6.05 *	3.33 *
	*SD*	3.00	1.46	1.64	1.31	3.14	1.60
Decision time	*M*	15.64	7.01	15.12	7.50	15.32	6.73
	*SD*	7.26	6.07	6.28	5.82	7.28	6.33
Scan path ratio	*M*	6.28	5.19	6.06	6.06	7.93	5.22
	*SD*	2.48	4.20	3.24	4.76	9.59	2.93

Note. All measures are for target-present trials. Averages were computed within participants, then across participants. Statistically significant figures reported here and below are marked with an asterisk (group differed from the other groups, *p* < 0.05); viewing condition was significant for all analyzed measures and is not marked. Accuracy and scan path ratio are descriptive only. SA = saccadic amplitude; RT = reaction time; TTFF = time to first fixation.

## Data Availability

Data supporting reported results will be made available upon reasonable request.

## Abbreviations

The following abbreviations are used in this manuscript:

HVP	Holistic Visual Processing
GCV	Gaze-Contingent Viewing
SA	Saccadic Amplitude
GAM	Generalized Additive Model
TTFF	Time to First Fixation
